# Na_V_1.7, 1.8 and 1.9 mutations linked to painful channelopathies have no pain phenotype in UK Biobank

**DOI:** 10.1093/braincomms/fcag374

**Published:** 2026-10-07

**Authors:** Graeme W T Newton, Corin Charman, Aidan P Nickerson, Keith G Phillips, Achim Kless, James P Dunham, Anthony E Pickering

**Affiliations:** Anaesthesia, Pain, and Critical Care Sciences, School of Physiology, Pharmacology, & Neuroscience, University of Bristol, Bristol BS8 1TD, UK; Newton Research, Kirkwall KW15 1JQ, UK; Anaesthesia, Pain, and Critical Care Sciences, School of Physiology, Pharmacology, & Neuroscience, University of Bristol, Bristol BS8 1TD, UK; Eli Lilly & Company, Bracknell RG12 1PU, UK; Anaesthesia, Pain, and Critical Care Sciences, School of Physiology, Pharmacology, & Neuroscience, University of Bristol, Bristol BS8 1TD, UK; Eli Lilly & Company, Bracknell RG12 1PU, UK; Anaesthesia, Pain, and Critical Care Sciences, School of Physiology, Pharmacology, & Neuroscience, University of Bristol, Bristol BS8 1TD, UK; Grünenthal GmbH, Aachen 502099, Germany; Eli Lilly & Company, Bracknell RG12 1PU, UK; Anaesthesia, Pain, and Critical Care Sciences, School of Physiology, Pharmacology, & Neuroscience, University of Bristol, Bristol BS8 1TD, UK; Anaesthesia, Pain, and Critical Care Sciences, School of Physiology, Pharmacology, & Neuroscience, University of Bristol, Bristol BS8 1TD, UK

**Keywords:** pain, genetics, Na_V_1.7, Na_V_1.8, Na_V_1.9

## Abstract

The voltage-gated sodium channels Na_V_1.7, Na_V_1.8 and Na_V_1.9, encoded by the *SCN9A*, *SCN10A* and *SCN11A* genes, respectively, are integral to nociceptor excitability and pain sensation. Multiple gain-of-function mutations in these ion channels have been reported to cause autosomal dominant painful channelopathies, including primary erythromelalgia, paroxysmal extreme pain disorder and familial episodic pain. Conversely, autosomal recessive loss-of-function mutations in Na_V_1.7 have been reported to cause painless channelopathies. The prevalence and impact on carriers of these voltage-gated sodium channel genetic variants in the wider population has yet to be explored. A combined genetic database and literature search was performed to identify pathogenic gain-of-function variants linked to painful channelopathies which had supporting *in vitro* electrophysiological evidence. Loss-of-function variants were identified by their inferred impact upon protein sequence. We investigated these variants within the UK Biobank cohort 500k whole-exome sequencing dataset. Logistic regression was used to examine whether carrying these variants was associated with chronic pain, neuropathic pain, and analgesic or anti-neuropathic drug prescriptions (adjusting for age, sex and 10 genetic principal components). For Na_V_1.7, 20/59 putative pathogenic gain-of-function mutations identified from literature were found in UK Biobank, with 148 901 carriers (>29% of the cohort). For Na_V_1.8, 4/5 gain-of-function mutations were found, with 1104 carriers. For Na_V_1.9, 13/17 gain-of-function mutations were found, with 1324 carriers. For predicted loss-of-function mutations in Na_V_1.7, Na_V_1.8 and Na_V_1.9, there were 723, 1307 and 1143 heterozygous carriers, respectively, and 2 homozygous Na_V_1.9 loss-of-function carriers. A total of 21 Na_V_ mutation groups had ≥50 heterozygous carriers allowing logistic regression analysis. None of these groups had a significantly altered risk of chronic or neuropathic pain. Similarly, no differences in prescriptions of opioid analgesics or anti-neuropathic pain medications were found. Subsequent pathogenicity prediction indicated that the gain-of-function variants called in UK Biobank had significantly lower predicted pathogenicity scores than those that were absent from the cohort. These findings show that over one-third of the previously reported pathogenic Na_V_1.7 gain-of-function mutations are likely to be benign for pain. This study challenges the previously assumed high penetrance of these Na_V_ gain-of-function mutations for chronic pain, and we make recommendations to optimize the evidence-based criteria for genetic association studies investigating the role of ion channel variants in the pathogenesis of pain.

## Teaser

Newton *et al.* report that many previously described pathogenic Na_V_1.7, Na_V_1.8 and Na_V_1.9 variants are common and are not associated with chronic or neuropathic pain or analgesic use in the UK Biobank cohort. These findings indicate that stricter evidence standards in gain-of-function painful channelopathy studies may be required.

## Graphical Abstract

**Graphical Abstract:**
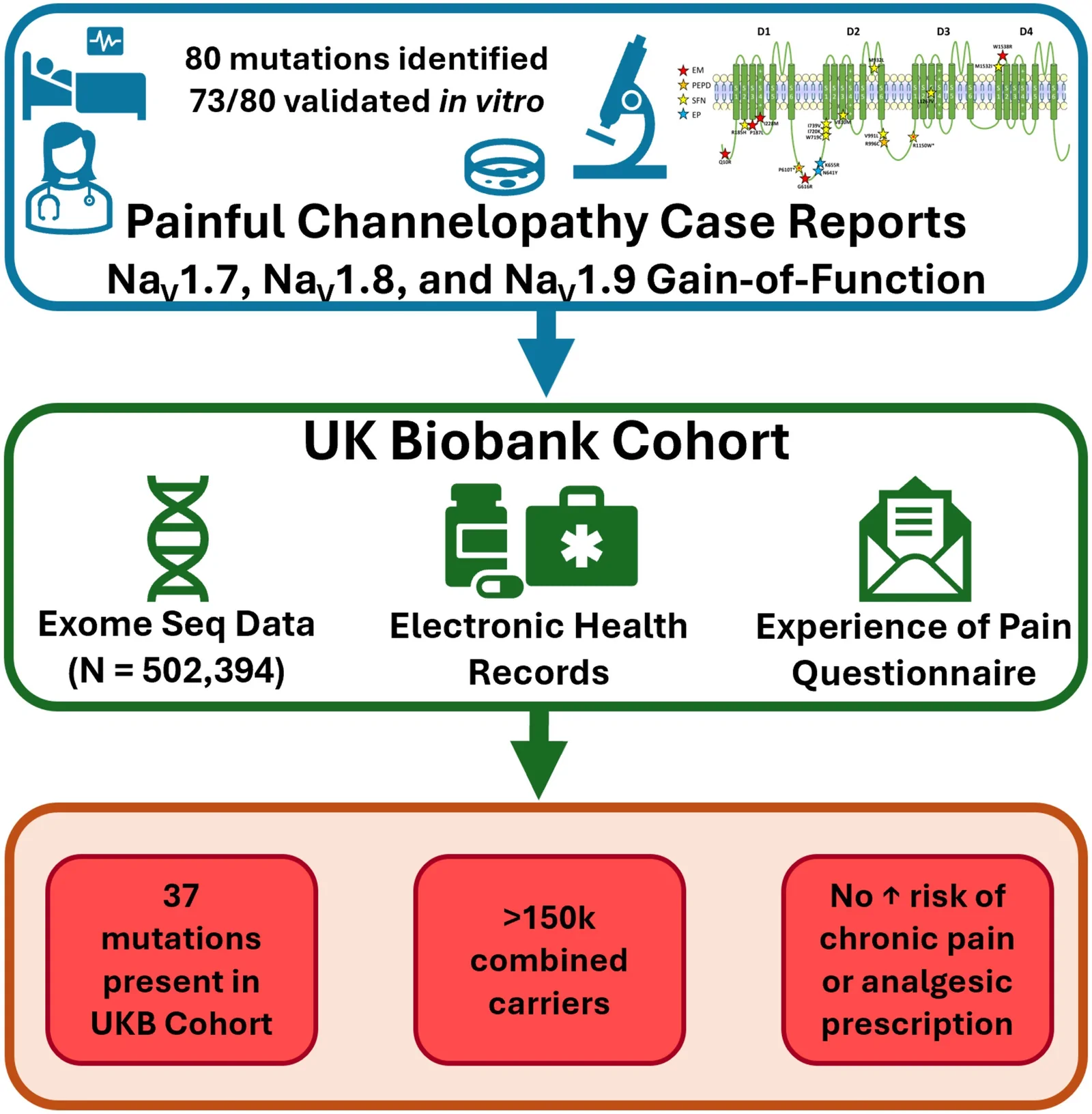

## Introduction

The voltage-gated sodium channels (VGSCs) Na_V_1.7, Na_V_1.8 and Na_V_1.9 play a crucial role in determining neuronal excitability in the peripheral nervous system and they have all been implicated in chronic pain, including inherited and acquired pain conditions.^1^ The alpha subunits of these VGSCs are encoded by the *SCN9A*, *SCN10A* and *SCN11A* genes. The influx of Na^+^ through VGSCs is a crucial determinant of neuronal excitability through the generation of action potentials.^2^ Herein, when we refer to mutations in VGSCs Na_V_1.7, Na_V_1.8 and Na_V_1.9, we are referring to amino acid changes in the protein of the alpha subunit.

The VGSC alpha subunit has four domains, each with six transmembrane segments and a pore-forming loop.^3^ The fourth transmembrane segment (S4) in each domain acts as a voltage sensor. The fifth and sixth transmembrane segments (S5 and S6) form the channel pore. The link between the third and fourth domains forms the fast inactivation gate, which blocks the channel pore after depolarization, preventing further sodium influx (Fig. 1A).^3^

**Figure 1:**
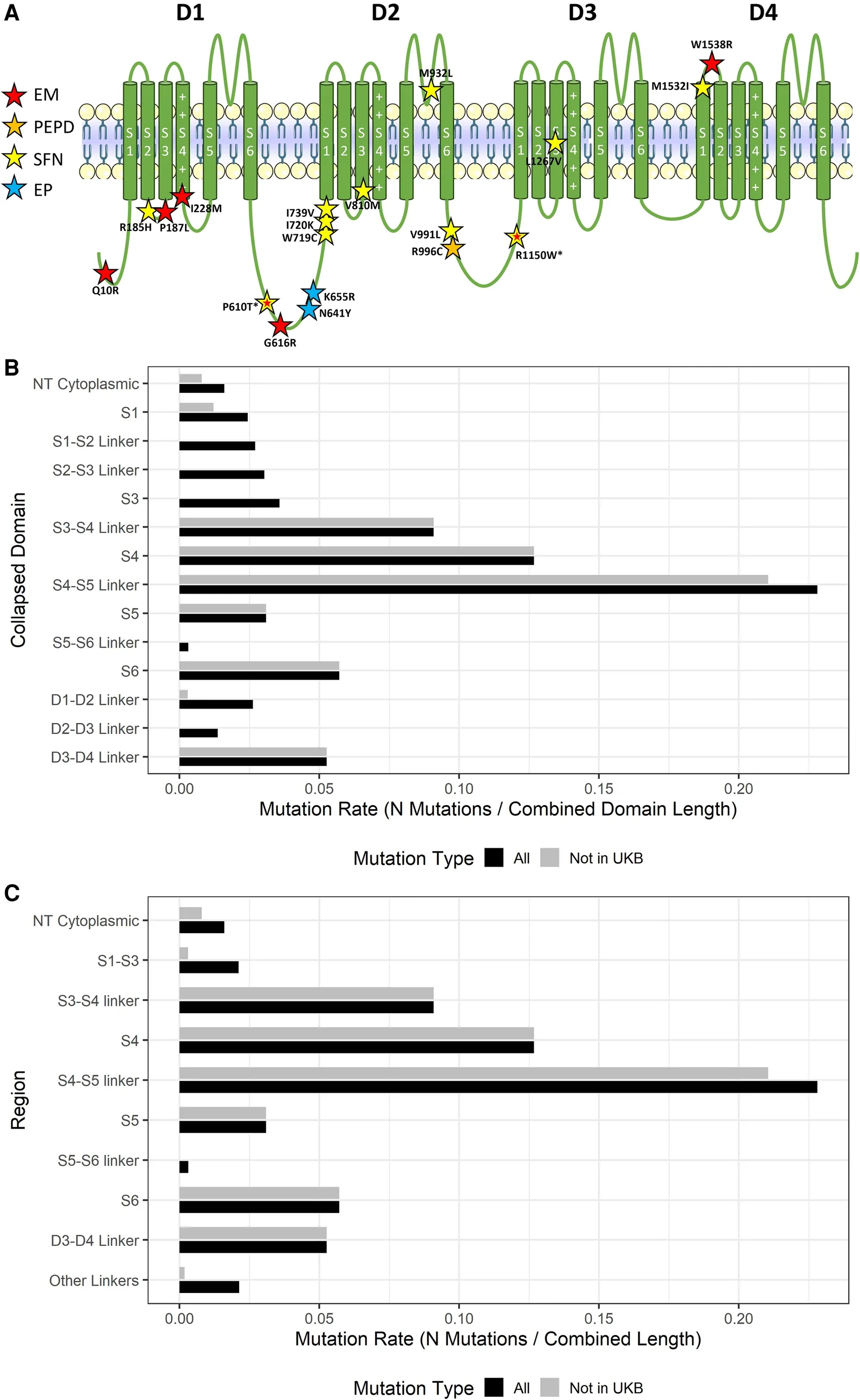
**Distribution of Na_V_1.7 channelopathy mutations.** (**A**) Schematic of Na_V_1.7 protein annotated with mutations present in UK Biobank identified with at least one heterozygous carrier. EM = erythromelalgia, PEPD = paroxysmal extreme pain disorder, SFN = small-fibre neuropathy, EP = epilepsy. *Mutation initially described in erythromelalgia patients but later considered to be a SFN mutation. **(B)** Distribution of all known Na_V_1.7 (*N* = 58) gain-of-function channelopathy mutations versus just the subset not found in UK Biobank collapsed by segment across domains and expressed as mutation rate (number of mutations/length of segments combined). **(C)** Distribution of the same Na_V_1.7 gain-of-function channelopathy mutations (*N* = 58) collapsed in a manner similar to Brunklaus *et al*.^4^ NT = N-terminus, D = domain, S = segment, other linkers = D1–D2 + D2–D3 Linkers.

In primary afferent nociceptors, Na_V_1.7 is considered a threshold channel which is crucial for the initiation of action potentials in response to generator potentials.^1^ Na_V_1.7 has been causally linked to multiple human pain-related channelopathies,^1^ supporting its critical role in nociception. In congenital insensitivity to pain (CIP), homozygous Na_V_1.7 mutations prevent nociceptor firing.^5^ These patients suffer repeated injuries due to loss of pain sensation, including fractures, Charcot joints and ulceration, consequently having a greatly shortened life expectancy.

Gain-of-function Na_V_1.7 mutations have been associated with rare inherited chronic pain conditions such as primary erythromelalgia and paroxysmal extreme pain disorder (PEPD), which can occur with a single heterozygous genetic variant. Patients with primary erythromelalgia typically present in early childhood and suffer from extreme pain that is refractory to analgesics or anti-neuropathic pain medications.^6,7^ It can be inherited in an autosomal dominant pattern or can occur *de novo* with new Na_V_1.7 mutations in individuals without a family history.^8^ Many unique Na_V_1.7 gain-of-function mutations have been causally linked to erythromelalgia.^1^ There appears to be an association between disease presentation and the biophysical properties of the mutant Na_V_1.7 channels with more severe electrophysiological changes linked to earlier disease onset.^9,10^

PEPD was first reported under the term ‘familial rectal pain’ as the index infant presented with episodic rectal, ocular, and mandibular pain and redness in the affected areas.^11^ The condition was renamed PEPD to reflect its clinical variability.^12^ PEPD is comparatively rarer than erythromelalgia and is consequently less well phenotyped.^13^ Gene linkage studies initially tied PEPD to chromosome 2 and, given the similarities in episodic burning pain and redness, the ‘erythromelalgia gene’, *SCN9A*, was sequenced.^12^ This identified gain-of-function Na_V_1.7 mutations in these PEPD patients and again identified both autosomal dominant inheritance patterns and *de novo* mutations. Small fibre neuropathy (SFN) is typically an acquired disease which can be a cause of chronic pain. Diabetes, toxin exposure and immune disorders are common causes of SFN, though many cases are idiopathic. There is increasing evidence for a genetic component in both idiopathic SFN and secondary SFN.^14^ Recent studies have reported associations with a number of Na_V_1.7 mutations which are more prevalent in cohorts of SFN patients.^15,16^

Functional studies of Na_V_1.7 have been performed on many mutations identified in patients with erythromelalgia, PEPD and SFN. These are typically patch clamp studies in heterologous cell lines. Na_V_1.7 mutations associated with erythromelalgia are usually characterized by a hyperpolarising shift in activation, decreasing the action potential threshold. In contrast, Na_V_1.7 mutations linked to PEPD frequently result in a depolarizing shift in fast inactivation.^17^ Spontaneous activity and reduced activity-dependent slowing of C-fibres were identified with microneurography in an erythromelalgia patient carrying the I228M mutation.^18^ Microneurography in another erythromelalgia patient with the Na_V_1.7 I848T mutation showed C-fibres exhibited aberrant responses to electrical stimulation.^18^

This cumulative weight of genetic and functional evidence linking Na_V_1.7 to inherited pain conditions is well accepted by the field.^1,17,19^ This evidence supported the rationale for development of analgesics targeting Na_V_1.7, with three small molecule inhibitors completing phase I/II trials.^20,21^ Although Na_V_1.7 has been a challenging target, in part due to the structural conservation of VGSCs and autonomic side effects like hypotension, there are active industry programmes developing small molecule and using RNA therapeutics to alter protein expression.^22^

Of the other VGSCs expressed in peripheral neurons, Na_V_1.8 contributes to the rising phase of action potentials.^1^ Gain-of-function mutations in Na_V_1.8 have been proposed as causal for SFN and there has been a single case report of an erythromelalgia patient.^23-25^ Loss-of-function mutations have been reported, but not in the context of pain.^26,27^ A selective Na_V_1.8 inhibitor, suzetrigine, was approved by the FDA in 2025 as an analgesic for moderate-to-severe acute pain consistent with its key role in nociceptive processing.^28^ The function of Na_V_1.9 is as a subthreshold channel that amplifies generator potentials.^1^ It is a less well characterized VGSC in part due to difficulty in electrophysiological recordings of its conductance.^29^ Gain-of-function mutations in Na_V_1.9 have been causally linked to SFN, familial episodic pain syndrome (FEPS), erythromelalgia and CIP.^30-34^

Studies of these putative pathogenic Na_V_ mutations have been conducted in the setting of rare inherited conditions or painful SFN.^1^ The prevalence of these Na_V_ mutations in the wider general population and their potential association with pain phenotypes has not been systematically examined as the identification of these rare genetic variants in large population cohorts was previously not feasible—a situation that has now changed with increased availability of whole-exome or whole-genome sequencing.

In this study, the UK Biobank (UKB) 500k whole-exome sequencing dataset is explored to identify participants carrying validated, painful, gain-of-function or predicted loss-of-function VGSC genetic variants and probe their association with pain phenotypes, such as chronic and neuropathic pain, and analgesic and anti-neuropathic medication prescription. We also explore the relationship between phenotypes and Na_V_ mutation evolutionary conservation, pathogenicity predictions and presence of paralogues causing other inherited diseases.

## Materials and methods

### Identification of pathogenic Na_V_1.7, Na_V_1.8 and Na_V_1.9 mutations

Mutations linked to painful channelopathies in the Na_V_1.7, Na_V_1.8 and Na_V_1.9 proteins were identified from UniProt.^35^ In addition, PubMed was searched for the primary research articles associated with these mutations using the term ‘SCN9A OR NaV1.7 AND *protein mutation name*’. This primary literature and reviews of Na_V_ channelopathy mutations were also consulted as part of this process with snowball searches of the cited literature.^1,36-39^

Mutations reported in the primary literature as causal for erythromelalgia, PEPD and FEPS, were included, both with and without electrophysiological gain-of-function evidence. Mutations reported as being associated with idiopathic SFN were included only if there was gain-of-function electrophysiological evidence. This was because several SFN cohort studies have reported an increased prevalence of multiple proposed gain-of-function VGSC mutations, but without functional testing.^40,41^ An SFN-associated Na_V_ mutation was considered to have an experimentally validated gain-of-function if there was a statistically significant change in any of the following: activation threshold, slow inactivation, fast inactivation or resting membrane potential.

Two additional Na_V_1.7 mutations, N641Y and K655R, were reported to be causal for epilepsy and had gain-of-function electrophysiological evidence.^42^ These mutations were included to assess whether this also translated into a pain phenotype.

### Pathogenicity predictions

We obtained pathogenicity predictions for all Na_V_ mutations from UniProt, Combined Annotation Dependent Depletion (CADD), Evolutionary model of Variant Effect (EVE) and AlphaMissense. UniProt is a public archive of protein sequence and annotation data from manually and computer-curated datasets.^35^ CADD scores are produced by a machine learning model that includes >60 input parameters spanning evolutionary conservation to tissue expression and are scored at the variant level.^43^ The scores are then ranked and scaled between 0 and 99, with higher scores meaning a variant is likely more deleterious. The scaling is logarithmic, a score >10 meaning the mutation is in the top 10%. The authors suggest a threshold of 15 for identifying potentially pathogenic variants (top 5%). The EVE score is generated by a machine learning model that predicts pathogenicity based on amino acid sequence data from >140k species—it scores between 0 and 1, and the proposed pathogenicity threshold is 0.7.^44^ AlphaMissense is a machine learning model that predicts the pathogenicity of missense mutations based on the conservation of protein sequence, rarity and structural context. Like EVE, it scores between 0 and 1, and the predicted pathogenicity threshold is 0.7.^45^ EVE and AlphaMissense scores are on the protein mutation level.

### Protein sequence alignment and identification of paralogs

Conserved amino acids in an ion channel protein sequence are more likely to be essential for function, therefore mutations affecting these sites are more likely to be deleterious.^46^ Multiple sequence alignment was performed to assess the degree of conservation for individual amino acid residues. Protein sequences of Na_V_1.7–1.9 from human (*Homo sapiens*), chimpanzee (*Pan troglodytes*), rhesus macaque (*Macaca mulatta*), cow (*Bos taurus*), pig (*Sus scrofa*), dog (*Canis lupus familiaris*), rabbit (*Oryctolagus cuniculus*), guinea pig (*Cavia porcellus*), rat (*Rattus norvegicus*) and mouse (*Mus musculus*) were downloaded from UniProt for interspecies sequence alignment. Protein sequences of the human Na_V_1.1–1.9 and Na_X_ channels were downloaded from UniProt for intraspecies sequence alignment. Sequence alignment used the MUSCLE algorithm in Jalview.^47,48^ Sequence alignment scores are presented as integers from 0 to 10, representing the number of species, or Na_V_ channels, where this amino acid was conserved.

Another method to triangulate the predictions of pathogenicity is through the comparative analysis of paralogues of each mutation in the other human VGSC, where there is strong sequence conservation in the functionally important domains.^4^ Each pathogenic mutation was evaluated for equivalent mutations in the other VGSCs that had been linked to other diseases, such as epilepsy or cardiac conduction deficits, and whether these had been shown to cause a gain-of-function (https://scn-viewer.broadinstitute.org/).

### UK Biobank cohort

UKB cohort contains 502 394 participants (54.4% female), aged 37–73 years at enrolment (2006–2010).^49^ The age range at the time of this study is 52–89. Participants have undergone genetic sequencing, detailed phenotypic screening, and had their NHS hospital inpatient and prescription data linked. In 2022, the final whole exome sequencing dataset for UKB was released.^50^

#### Sequencing data processing and genetic variant annotation

The UKB Research Analysis Platform (RAP) was used to perform analysis of whole exome sequencing data. The *SCN9A*, *SCN10A* and *SCN11A* gene regions were extracted using PLINK 2 (www.cog-genomics.org/plink/2.0/). Variant calls were filtered to only sites where ≥90% of individuals had sequencing depth >10, using the pre-calculated ‘90pct10dp_qc_variants.txt’ helper files. PLINK2 was used to calculate the number of heterozygous and homozygous minor allele calls and convert the sequencing data to the VCF format.^51^ Ensembl Variant Effect Predictor (release 113.0) was used to annotate VCF files.^52^ Heterozygous carriers of variants annotated as frameshift, stop gain, splice donor, splice acceptor, start lost, or transcript ablation were classified as predicted loss-of-function and grouped together into a combined loss-of-function group. Two homozygous *SCN11A* loss-of-function carriers were identified and excluded from the *SCN11A* model.

#### Pain phenotype categorization

The UKB RAP was used to extract the phenotype data. Seven binary phenotypes were generated using a combination of data from the original assessment at the cohort’s inception, the Experience of Pain Questionnaire (EoPQ) and prescription history from the electronic health record linkage.^53^ In the original recruitment assessment, participants were considered positive for the ‘chronic pain’ phenotype if they had pain for more than 3 months in any specific body area or more generally (Supplementary Table 1). A participant was considered positive for the ‘neuropathic pain’ phenotype if they scored 3 or greater, in the seven questions derived from the DN4 in the EoPQ which was completed in 2019 (Supplementary Table 2).^54^ This phenotype has already been used to explore the epidemiology of neuropathic pain in the UKB cohort.^53^

To extract analgesic and anti-neuropathic pain prescription phenotypes, 56 million prescription events between 1986 and 2018 of 222 065 participants were queried using PySpark in an Apache Spark cluster. Regular expressions extracted all drug entries regardless of dose or frequency (Supplementary Table 3). A participant was considered positive for a prescription drug phenotype if they had ever been prescribed a drug within that class. For example, if a participant had been prescribed carbamazepine, they were considered positive for the ‘*NaV_blocker’* phenotype. The prevalence of participants having ever had prescriptions for Na_V_ channel blockers was 3.1% (4928); gabapentinoids, 10.0% (16 125); tricyclic antidepressants (TCAs), 26.0% (41 990); strong opioids, 5.5% (8878); and dual-mechanism opioids, 18.0% (29 090).

### Statistical analysis

The primary objective was to investigate the association between specific gain- or loss-of-function mutations and the likelihood of experiencing chronic pain, neuropathic pain, or receiving analgesic and anti-neuropathic drug prescriptions. Binomial logistic regression models were used to examine the relationships between the outcome and predictor variables while adjusting for age, sex and the first 10 genetic principal components.^55^

Each gene was treated independently, and a participant was assigned to a mutation group (e.g. SCN9A_I228M) if they were a homozygous or heterozygous carrier of a gain-of-function mutation, or a heterozygous carrier of a predicted loss-of-function mutation. Mutation groups with <50 carriers were excluded from the regression analysis. Participants who carried multiple gain- or loss-of-function mutations (or a combination thereof) were removed from the primary analysis to isolate the effect of each mutation individually. The majority of the ‘multi-carriers’ was driven by the so-called ‘*Neanderthal’ SCN9A* haplotype (including the M932L and V991L mutations), which has been linked to slightly increased pain sensitivity (Supplementary Table 4).^56,57^

Participants with missing outcome data were excluded. The models were checked for multicollinearity. All logistic regression models were generated using ‘*glm()’* function from the base R package ‘*stats’* R version 4.5.0 and followed the same form, where *P* represented the probability of experiencing the phenotype being tested, and *β* represents model coefficients.


$$\begin{array}{rl} \log\;\left(\frac{p}{1-p}\right)= & \;\beta_{0}+\beta_{1}(mut.)+\beta_{2}(age)+\beta_{3}(sex)\\ & +\overset{14}{\underset{i=4}{\sum }}\;\beta_{i}(PC_{i-3}) \end{array}$$

*P* values were adjusted at the model level to correct for multiple testing (7 logistic regressions) using the false discovery rate (FDR) method as implemented in the ‘*p.adjust()’* function. The significance threshold was *P* < 0.05. An odds ratio (OR) > 1 indicated an increased likelihood of experiencing the phenotype. ORs are presented with 95% confidence intervals. Raw outputs from logistic regression models are presented in Supplementary Table 5.

Power calculations were performed using the ‘*powerLogisticBin()’* function from the ‘*powerMediation’* R package for logistic regression using sample sizes and prevalences of the chronic pain phenotype observed in the *SCN9A* model control population. Our power to detect an OR of 2 in a group of 50 carriers for the chronic pain phenotype was 68.7%. The minimum detectable OR for a group of 50 carriers with 80% power is 2.22, which would correspond to a chronic pain rate of 62.9% in the carrier groups versus 43.5% in the control group. Alternatively, the minimum sample size to detect an OR of 2 at 80% power was 66. Given the gain-of-function mutations are suggested to be highly penetrant (i.e. near 100% chronic pain prevalence), we would be adequately powered to detect a gain-of-function in our analysis.

## Results

A total of 80 unique putative gain-of-function Na_V_1.7, Na_V_1.8 and Na_V_1.9 mutations were identified from the literature search. Almost all the reported erythromelalgia mutations (36/39, 92%), PEPD mutations (9/12, 75%) and FEPS mutations (5/6, 83%) had a gain-of-function confirmed using electrophysiology. All 21 SFN mutations and 2 epilepsy mutations caused an electrophysiological gain-of-function *in vitro* (Supplementary Table 6). Na_V_1.7 contained the greatest number of unique putative pathogenic mutations (*N* = 58) that are dispersed widely throughout the Na_V_1.7 protein. Every domain (D1–4) contains pathogenic mutations, as well as all segments (S1–6) and the pore-forming loop (contained within the S5–S6 linker). Calculation of the mutation rate in each domain indicates that the S3–S4 linker, S4–S5 linker and S5 are relatively enriched for Na_V_ mutations (Fig. 1B). Collapsing these mutations into topological groupings, as per Brunklaus *et al*.,^4^ emphasizes this enrichment in the conserved functional domains (Fig. 1C).

### Many UK Biobank participants carry putative pathogenic VGSC mutations

We found 34% of putative pathogenic Na_V_1.7 mutations (20/58), 80% of putative pathogenic Na_V_1.8 mutations (4/5) and 75% of putative pathogenic Na_V_1.9 mutations (12/16) were carried by one or more participants in UKB (**Table 1**). Of the 36 mutations detected, 33 (92%) showed electrophysiological gain-of-function *in vitro*. In aggregate, a total of 150 510/502 137 UKB participants (30.0%) carried at least one of these putatively pathogenic VGSC mutations, and of these, 8823 were homozygous carriers (**Table 1**). There were 428 unique loss-of-function genetic variants, with a combined total of 3166 carriers (0.6%). There were two homozygous carriers of a loss-of-function mutation in Na_V_1.9 **(**Supplementary Table 7**)**.

**Table 1:** Number of carriers, minor allele frequency, electrophysiology gain-of-function evidence status and disease association from case report

Mutation	Het	Hom	UKB MAF	1000G MAF	gnomAD MAF	Ephys GoF	Disease
NaV1.7_Q10R	3	0	3.193 × 10^6^	0	7.473 × 10^5^	Y	EM
NaV1.7_R185H	1883	4	2.012 × 10^3^	0	2.813 × 10^3^	Y	SFN
NaV1.7_P187L	15	0	1.596 × 10^5^	0	1.795 × 10^5^	N	EM
NaV1.7_I228M	1381	1	1.472 × 10^3^	1.000 × 10^3^	9.175 × 10^4^	Y	EM
NaV1.7_P610T^a^	24 430	375	2.680 × 10^2^	3.280 × 10^2^	2.522 × 10^2^	Y	EM
NaV1.7_G616R	78	0	8.301 × 10^5^	0	1.127 × 10^4^	Y	EM
NaV1.7_N641Y	22	0	2.341 × 10^5^	0	2.689 × 10^5^	Y	EP
NaV1.7_K655R	2519	4	2.689 × 10^3^	3.000 × 10^3^	1.923 × 10^3^	Y	EP
NaV1.7_W719C^b^	189	1	2.011 × 10^4^	1.000 × 10^3^	9.110 × 10^4^	Y	SFN
NaV1.7_I720K	256	0	2.724 × 10^4^	2.000 × 10^3^	2.300 × 10^4^	Y	SFN
NaV1.7_I739V	3877	7	4.141 × 10^3^	3.000 × 10^3^	2.711 × 10^3^	Y	SFN
NaV1.7_V810M	215	2	2.331 × 10^4^	1.000 × 10^3^	2.102 × 10^4^	Y	SFN
NaV1.7_M932L	2644	22	2.861 × 10^3^	0	1.912 × 10^2^	Y	SFN
NaV1.7_V991L	2642	21	2.856 × 10^3^	0	1.683 × 10^2^	Y	EM
NaV1.7_R996C	15	0	1.596 × 10^5^	0	2.129 × 10^5^	N	PEPD
NaV1.7_R1150W^c^	108 583	8405	1.335 × 10^1^	1.262 × 10^1^	8.755 × 10^1^	Y	EM
NaV1.7_L1267V	2396	4	2.558 × 10^3^	2.000 × 10^3^	1.634 × 10^3^	Y	SFN
NaV1.7_M1532I	4	0	4.257 × 10^6^	0	8.979 × 10^6^	Y	SFN
NaV1.7_W1538R	2245	6	2.402 × 10^3^	0	2.375 × 10^3^	Y	EM
NaV1.8_L554P	28	0	2.980 × 10^5^	1.000 × 10^3^	9.256 × 10^5^	Y	SFN
NaV1.8_M650K	97	0	1.032 × 10^4^	0	5.185 × 10^4^	Y	EM
NaV1.8_A1304T	26	0	2.767 × 10^5^	0	4.866 × 10^5^	Y	SFN
NaV1.8_G1662S	951	2	1.016 × 10^3^	1.000 × 10^3^	1.361 × 10^3^	Y	SFN
NaV1.9_R222H	1	0	1.064 × 10^6^	0	1.591 × 10^6^	N	FEPS
NaV1.9_R222S	1	0	1.064 × 10^6^	0		Y	FEPS
NaV1.9_R225C	4	0	4.257 × 10^6^	0	5.122 × 10^6^	Y	FEPS
NaV1.9_I381T	31	0	3.299 × 10^5^	0	4.772 × 10^6^	Y	SFN
NaV1.9_K419N	458	0	4.874 × 10^4^	0	5.750 × 10^4^	Y	SFN
NaV1.9_A582T	66	0	7.024 × 10^5^	0	1.729 × 10^4^	Y	SFN
NaV1.9_A681D	3	0	3.193 × 10^6^	0		Y	SFN
NaV1.9_G699R	295	0	3.139 × 10^4^	0	1.614 × 10^4^	Y	SFN
NaV1.9_A842P	2	0	2.128 × 10^6^	0	1.590 × 10^5^	Y	SFN
NaV1.9_L1158P	149	0	1.586 × 10^4^	1.000 × 10^3^	5.270 × 10^4^	Y	SFN
NaV1.9_N1169S	263	0	2.799 × 10^4^	0	2.429 × 10^4^	Y	EM
NaV1.9_I1293V	18	0	1.916 × 10^5^	0	2.306 × 10^5^	Y	EM
NaV1.9_F1689L	33	0	3.512 × 10^5^	0	2.305 × 10^4^	Y	SFN

Please see Supplementary Table 8, which is an expanded version of this table that includes UniProt disease associations, machine learning pathogenicity prediction scores, and intra- and inter-species conservation for the putative pathogenic Na_V_1.7, Na_V_1.8 and Na_V_1.9 channelopathy mutations observed in UKB.

Het = number of heterozygous carriers, Hom = number of homozygous carriers, UKB = UK Biobank MAF, 1000G = 1000 Genomes Project MAF, gnomAD = gnomAD 4.1 non-UKB MAF, Ephys GoF = electrophysiological gain-of-function status. EM = erythromelalgia, PEPD = paroxysmal extreme pain disorder, EP = epilepsy, SFN = small fibre neuropathy.

^a^Originally proposed as an EM mutation but now considered to be SFN.

^b^Mutation caused by two unique genomic variants.

^c^Mutation not considered as pathogenic for this analysis due to commonality.

The allele frequencies calculated from the UKB whole exome sequencing dataset were similar to those previously reported in the 1000 Genomes Project and gnomAD 4.1.0 excluding UKB data (**Table 1**). All but two of the variants had a minor allele frequency (MAF) < 0.01 and are considered to be rare variants.^58^ The variants causing the Na_V_1.7 R1150W and P610T mutations had MAFs of 0.15 and 0.028, respectively. The commonality of the R1150W mutation, found in 116 988 participants, suggests it is extremely unlikely to be responsible for a highly penetrant pain phenotype. A sensitivity analysis supported this conclusion as a combined group of hetero- and homozygous carriers of R1150W do not have an increased risk of chronic pain (Fisher’s exact test, OR = 1.00[0.98–1.01], *P* = 0.5). The commonality of R1150W posed issues for downstream analysis, as participants carrying >1 mutation were excluded. Therefore, R1150W carrier status was not further considered during this analysis.

For the binomial regression analysis, only mutations that had ≥50 carriers after exclusion of multi-carriers and a gene-level heterozygous predicted loss-of-function group were assessed. The detailed results for three of the Na_V_1.7 mutations, selected as they had the strongest supporting evidence published for pathogenicity, and one Na_V_1.9 mutation are explored below.

### No pain phenotype in carriers of Na_V_1.7^I228M^

Na_V_1.7^I228M^ is one of the most extensively studied painful channelopathy mutations. Initially reported in three patients across two families, each had a presentation consistent with SFN—one who had features characteristic of erythromelalgia.^59^ An independent group reported this mutation in another erythromelalgia patient.^60^ Microneurography showed that this patient had spontaneous activity in their C-fibres and abnormal activity-dependent slowing.^18^ Whole-cell voltage clamp in HEK293 cells transfected with Na_V_1.7^I228M^ revealed a significant depolarizing shift in slow inactivation of the sodium conductance.^59^ Current clamp recordings in cultured rat trigeminal and dorsal root ganglion neurons transfected with Na_V_1.7^I228M^ showed a depolarizing shift in resting membrane potential, reduced spike threshold and spontaneous firing. Cultured DRG neurons expressing Na_V_1.7^I228M^ have significantly reduced neurite outgrowth, mitochondrial size and density.^61,62^  *In vivo*, a genetically modified mouse homozygous for Na_V_1.7^I228M^ develops skin lesions, an insensitivity to noxious hot and cold, and to the pruritogen chloroquine accompanied by a pronounced decrease in the expression of marker genes for C-fibre low-threshold mechanoreceptive neurons.^63^

The amino acid mutated in I228 is a highly conserved residue across species (10/10) and within the human Na_V_ family (8/10). UniProt categorized it as benign, however, both AlphaMissense and CADD predict I228M to be pathogenic, whereas EVE reports it as ambiguous (**Table 1**). Considering these predictions alongside the evidence base for the functional effects of this mutation, it was anticipated that I228M would be associated with a penetrant pain phenotype.

There are 1381 heterozygous carriers and 1 homozygous carrier of the I228M mutation in UKB (MAF = 0.001). The I228M mutation was not significantly associated with chronic pain (OR = 0.97[0.87–1.08], *P* = 0.98, *N* = 1330) or neuropathic pain (OR = 1.04[0.74–1.45], *P* = 0.98, *N* = 202). Similarly, there was no significantly increased prevalence of prescription of analgesic or anti-neuropathic pain medications (Fig. 2A).

**Figure 2:**
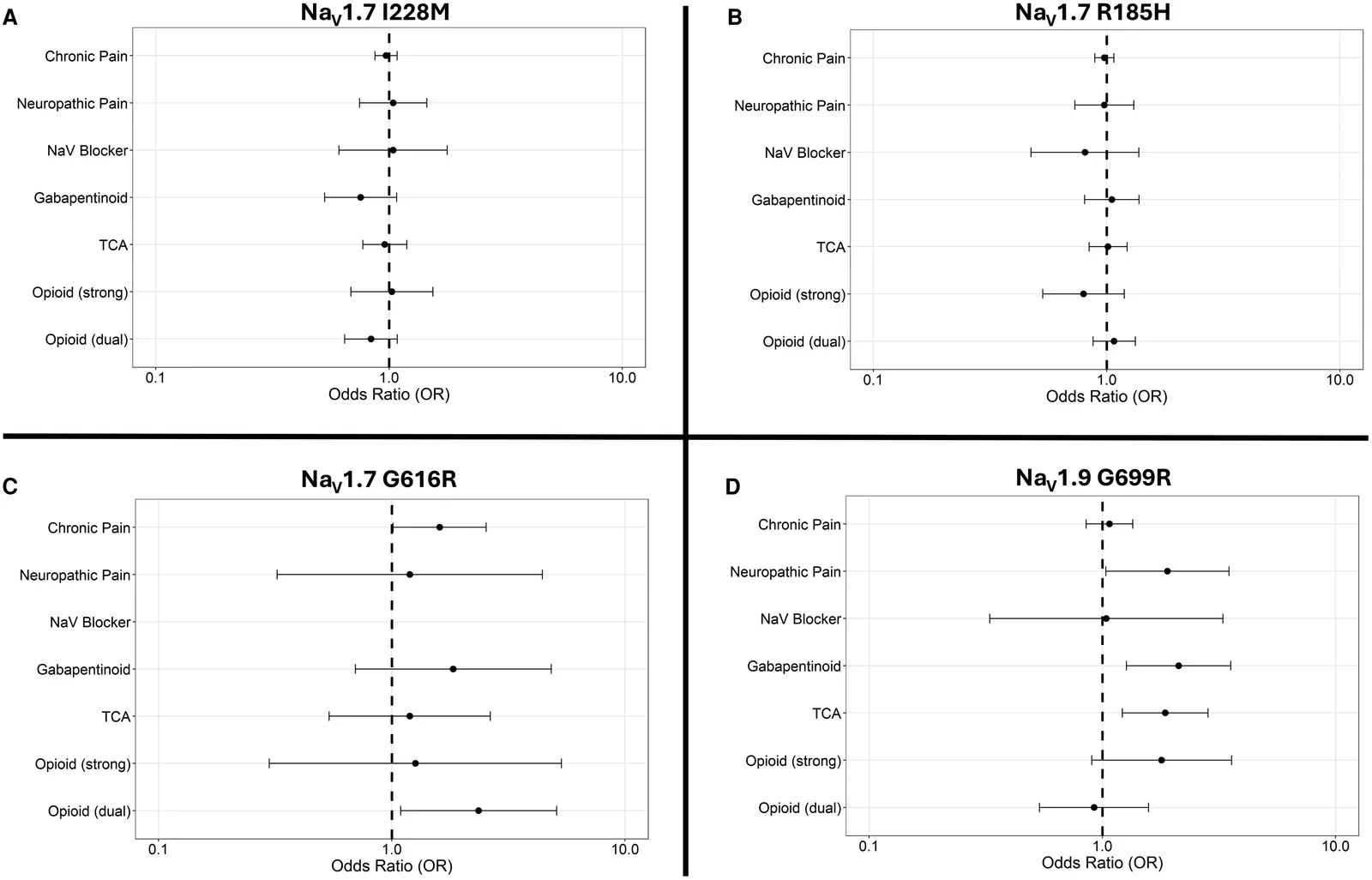
**Associations of representative channelopathy mutations with pain phenotypes in the UK Biobank cohort.** Na_V_1.7 I228M (*N* = 1330) **(A)**, Na_V_1.7 R185H (*N* = 1820) **(B)** and Na_V_1.7 G616R (*N* = 74) **(C)**, are not associated with an increased risk of any pain phenotype. Na_V_1.9 G699R (*N* = 95) (**D**) shows a hint of a signal for neuropathic pain but this does not survive correction for multiple comparisons. Odds ratios (ORs) for each phenotype are presented with their 95% confidence intervals. TCA = tricyclic antidepressant.

### No pain phenotype in carriers of Na_V_1.7^R185H^

The R185H mutation was first reported in two unrelated patients with SFN.^64^ The same mutation has also been identified in two siblings with epilepsy and febrile seizures.^65^ Recordings in mouse DRG neurons transfected with Na_V_1.7^R185H^ revealed a 35% reduction in current threshold for action potential discharge perhaps related to an increase in resurgent currents.^66^ An *SCN9A^R185H^* mouse line has been generated which exhibits thermal and mechanical hyperalgesia, and clonidine-induced place preference suggesting a spontaneous pain phenotype.^67^

The Na_V_1.7 R185 residue was highly conserved across species (10/10) and within the human Na_V_ protein family (9/10). UniProt categorized it as benign. The EVE and CADD models predicted R185H to be pathogenic whereas AlphaMissense reported it as ambiguous (**Table 1**).

There are 1883 heterozygous and 4 homozygous carriers of Na_V_1.7^R185H^ in UKB (MAF=0.002). Na_V_1.7^R185H^ was not associated with an increased risk of chronic pain (OR = 0.98[0.89–1.07], *P* = 0.98, *N* = 1820) or neuropathic pain (OR = 0.98[0.73–1.31], *P* = 0.98*, N* = *272*). Similarly, Na_V_1.7^R185H^ was not associated with a significantly increased prevalence of prescriptions for analgesics or anti-neuropathic pain medications (Fig. 2B).

### No pain phenotype in carriers of Na_V_1.7^G616R^

Na_V_1.7^G616R^ was initially observed in four erythromelalgia patients from the same family, across three generations, segregating with the phenotype in four of the five mutation carriers.^68^ In transfected mouse DRG neurons, Na_V_1.7^G616R^ currents had a depolarizing shift in inactivation and spontaneous firing in 33% of neurons.^68^

The G616 residue was highly conserved across species (10/10) and was moderately conserved within the human Na_V_ protein family (6/10). UniProt categorized it as pathogenic, and it was predicted to be pathogenic by CADD, ambiguous by AlphaMissense, and benign by EVE (**Table 1**).

G616R is very rare with only 78 heterozygous carriers in UKB (MAF = 0.0001). It was not significantly associated with chronic pain (OR = 1.60[1.01–2.54], *P* = 0.49, *N* = 74) nor with neuropathic pain (OR = 1.19[0.32–4.43], *P* = 0.98, *N* = 12). It was not associated with an increased prevalence of prescriptions of analgesics or anti-neuropathic pain medications (Fig. 2C).

### There is insufficient evidence Na_V_1.9^G699R^ increases the risk of neuropathic pain

The Na_V_1.9^G699R^ mutation was identified in a patient with idiopathic small-fibre neuropathy.^69^ Whole-cell patch clamp revealed a large electrophysiological gain-of-function, shifting steady-state fast inactivation by 6.3 mV and hyperpolarising activation by −10.1 mV. Na_V_1.9^G699R^ transfected cells exhibited a reduced activation threshold and depolarized resting membrane potential.

The G699 residue is highly conserved across species (10/10) and within the human Na_V_ protein family (9/10). UniProt categorized it as pathogenic, and it was predicted to be pathogenic by CADD and EVE, and ambiguous by AlphaMissense (**Table 1**).

The Na_V_1.9^G699R^ mutation is rare with 295 heterozygous carriers in UKB (MAF=0.0003). It was not significantly associated with chronic pain (OR = 1.07[0.85–1.35], *P* = 0.98, *N* = 294). There was a numerical trend towards an increased risk of neuropathic pain (OR = 1.90[1.03–3.49], *P* = 0.49, *N* = 46) and interestingly a similar trend towards an increased prescription of anti-neuropathic medications, TCAs (OR = 1.86[1.22–2.84], *P* = 0.20, *N* = 95) and gabapentinoids (OR = 1.80[0.90–3.58], *P* = 0.70, *N* = 95). While the unadjusted *P* values for these phenotypes were <0.05, none of these associations survived adjustment for multiple comparisons (Fig. 2D).

### No pain phenotype in carriers of any of the other Na_V_1.7, Na_V_1.8 or Na_V_1.9 mutations

The same analysis was applied to the other 17 Na_V_ mutation groups suggested to be pathogenic from the literature search and with >50 carriers in UKB and also for the three gene-level heterozygous predicted loss-of-function groups. No evidence was found of a significant association with chronic pain, prescription analgesics, or anti-neuropathic pain medications (Fig. 3).

**Figure 3:**
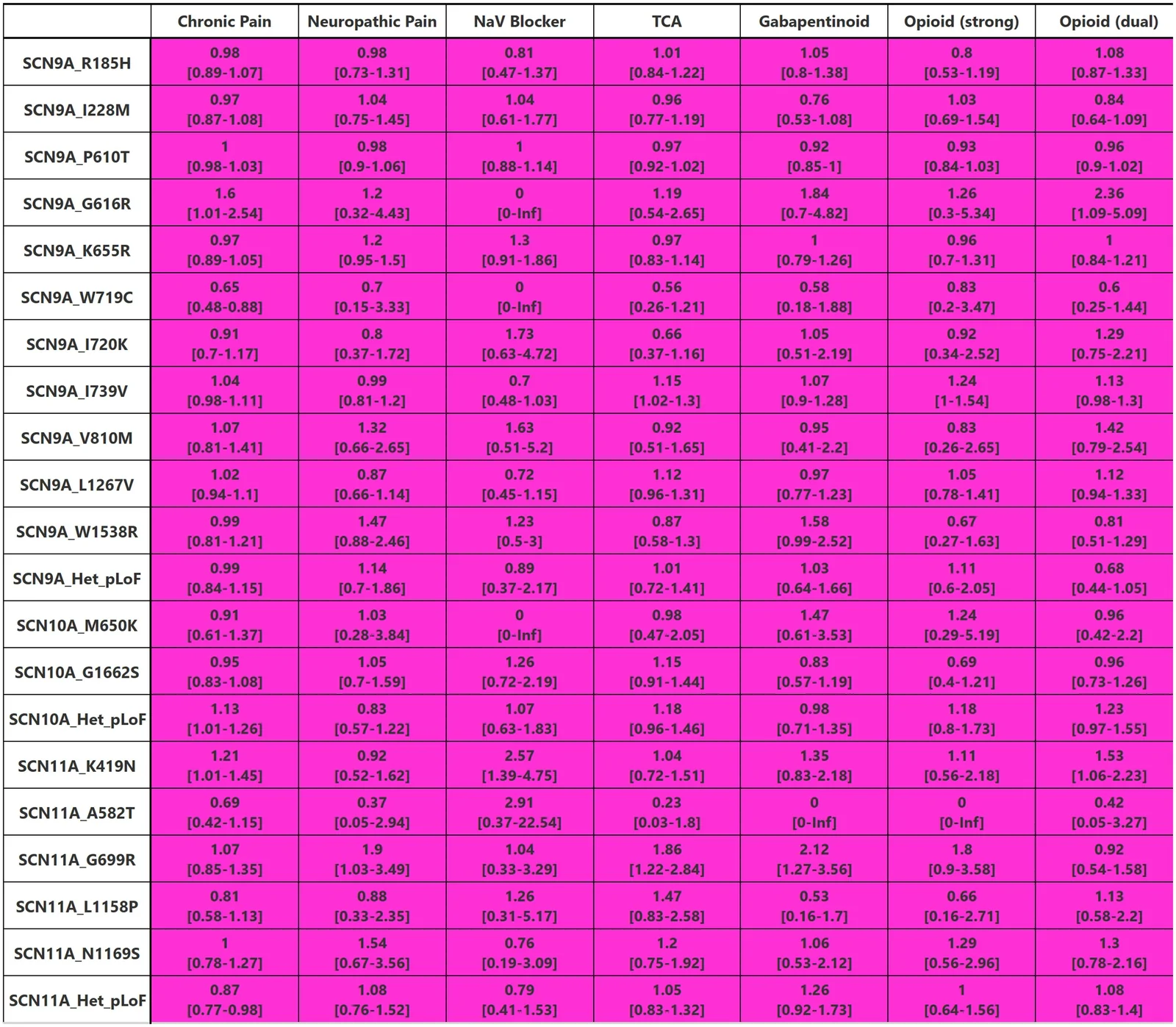
**Summary tile plot of all logistic regression analyses.** Tile plot of odds ratios (ORs) [±95% CI] across all genes and phenotypes, coloured by false detection rate (FDR)-adjusted *P* value (*P* < 0.05 = cyan, *P* > 0.05 = magenta). TCA = tricyclic antidepressant.

We aggregated all of the rarer mutations (<50 carriers in UKB) into a composite group for each gene and again found no evidence of a pain phenotype (Supplementary Table 9). As a further sensitivity analysis, we also aggregated all mutation carriers (including the rare mutations) together with participants carrying multiple mutations for each gene and found no evidence for an increased risk of chronic pain (Supplementary Table 9).

### Comparative pathogenicity predictions for Na_V_ mutations present or absent in UK Biobank

A grouped analysis of the pathogenicity predictions (assessed with CADD, AlphaMissense and EVE) for the mutations present in UKB compared to those which were absent showed that the absent mutations had a significantly greater predicted risk of pathogenicity (Fig. 4). Similarly, 32% of the mutations not present in UKB had paralogues with documented electrophysiological evidence of altered VGSC function whereas none of those present in UKB had any corroborating electrophysiological evidence (Supplementary Table 6). Almost all (91%) of the putative pathogenic Na_V_ mutations that were absent from UKB had paralogues that were associated with other inherited diseases whereas only a minority (36%) of the mutations observed in UKB had disease-associated paralogues. Re-plotting the distribution of the Na_V_1.7 mutations not in UKB showed that they were increasingly concentrated in the functionally important and conserved regions of the sodium channel protein (such as the S4, S5, S6 and D3–D4 linkers) as a direct consequence of winnowing out the mutations present in UKB that were mostly found in non-conserved regions like the D1–D2 and D2–D3 domain linkers (Fig. 1B). Collapsing the mutations into functional domains supported this conclusion (Fig. 1C).^4^

**Figure 4:**
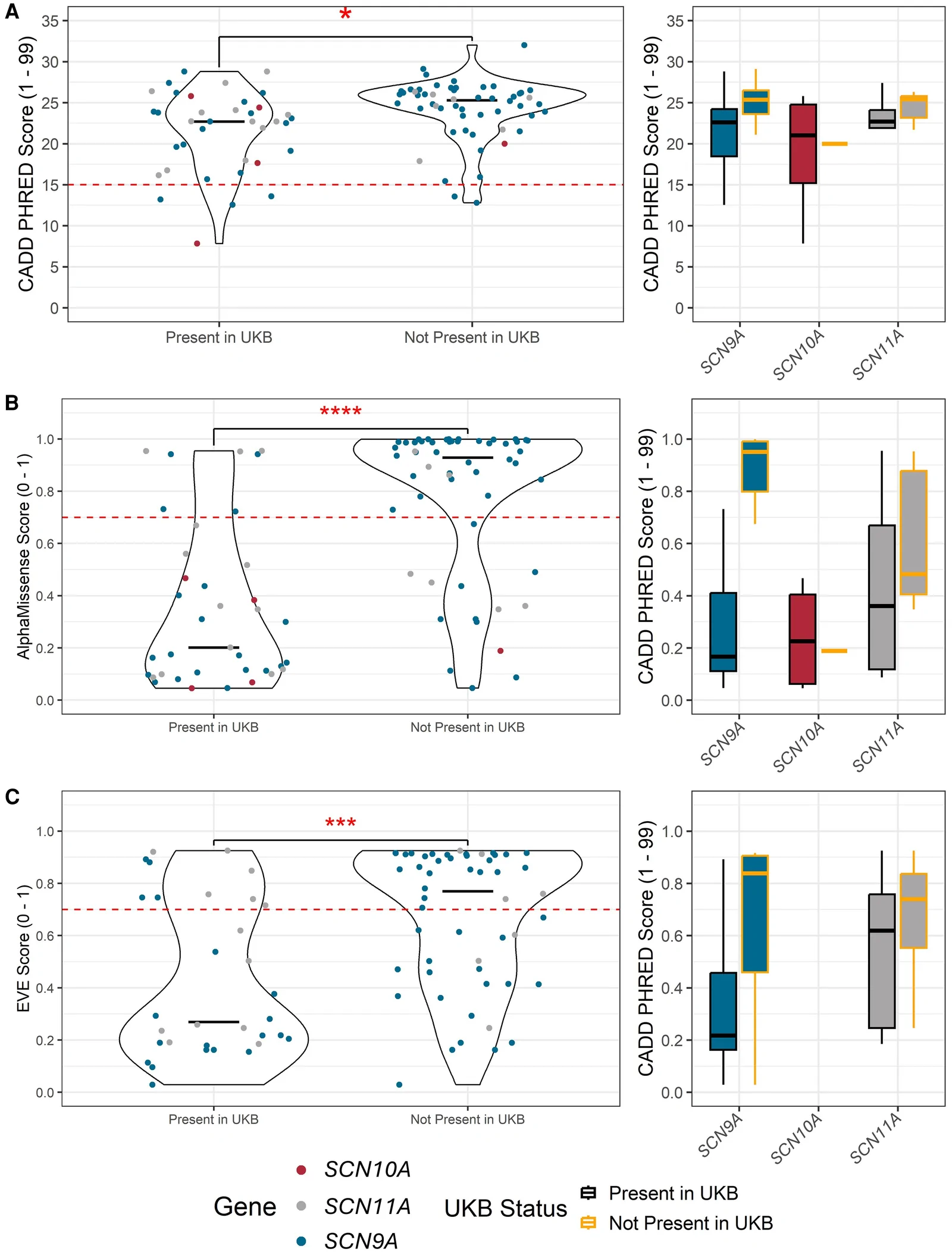
**Predicted pathogenicity scores of painful channelopathy mutations.** Violin plots of pathogenicity predictions from three machine learning models, CADD (**A**), AlphaMissense (**B**) and EVE (**C**), grouped by those present in UK Biobank dataset (*SCN9A N* = 20, *SCN10A N* = 4, *SCN11A N* = 13) or not present in UKB (*SCN9A N* = 46, *SCN10A N* = 1, *SCN11A N* = 7). Each point represents a single missense variant, coloured according to gene. Violin plots show the distribution of pathogenicity scores, with horizontal black bars indicating the median. Dashed red lines indicate the pathogenicity threshold for each prediction method. Asterisks denote statistical significance of the difference between UKB and non-UKB variants determined using a Wilcoxon rank-sum test (*P < 0.05; **P < 0.01; ***P < 0.001). Box plots (**D**) show CADD scores for each gene stratified by UKB status; boxes represent the interquartile range, the centre line indicates the median, whiskers extend to 1.5 × the interquartile range and error bars represent the standard error of the mean. All mutations present in UKB had significantly lower average pathogenicity scores than those not present in UKB (Wilcoxon signed-rank test; CADD: *W* = 631, *P* = 0.003; AlphaMissense: *W* = 324, *P* = 5.03 × 10^−8^; EVE: *W* = 453.5, *P* = 0.0005).

## Discussion

By leveraging the scale of the extensively-phenotyped UKB cohort and the availability of whole-exome sequencing data, we found that almost 45% of VGSC mutations previously reported to cause rare autosomal dominant inherited pain conditions or SFN were carried by almost 30% of this population sample. We expected to identify a highly penetrant pain phenotype given that almost all of these mutations had gain-of-function electrophysiological evidence, and in some cases even human *in vivo* evidence showing spontaneous C-fibre nociceptor firing. We found no evidence of a highly penetrant pain phenotype for multiple Na_V_1.7 mutations which have been reported as causal for erythromelalgia (I228M, G616R and W1538R), and which were expected to be strongly associated with pain.^10,59,60,68^ Similarly, there were no associations with pain for any of the SFN mutations. The remaining pathogenic mutations, including all PEPD variants, had few or no UKB carriers, possibly reflecting negative selection pressure.

From a historical perspective there has been a steady increase in the number of novel VGSC mutations associated with pain conditions following the original linkage of *SCN9A* variants to erythromelalgia.^8^ Early studies that identified the first erythromelalgia mutations used Sanger sequencing of the exons and flanking regions of the *SCN9A* gene to identify polymorphisms.^8,70^ Next was assessment of sequence conservation across species to determine functional importance.^71^ Then the commonality of the candidate variant was checked.^72^ An MAF of less than 1% is typically used for filtering potential candidate causal variants. In the absence of large population cohorts with genetic sequencing, some erythromelalgia studies sought to demonstrate the rarity of a variant by sequencing *SCN9A* in small ethnicity-matched control cohorts of 25–100 people.^70^ This combination of pain phenotype, familial co-segregation, sequence conservation and variant rarity has been used to infer pathogenicity.^8,73^ Several more recent studies have sequenced panels of 20–30 candidate ‘pain genes’.^60,74^ However, these approaches still suffer from confirmation bias by limiting the search to select genes.

Advances in sequencing technology have made exome sequencing more cost-efficient than targeted sequencing—reducing the risk of false-positive associations. Of the 58 reported gain-of-function pathogenic Na_V_1.7 mutations, only 2 (3%) were identified in studies that used whole-exome sequencing. The potential for inaccurate assignation of pathogenicity for the SFN mutations is likely greater and accordingly a higher proportion (74%) of these mutations is present in UKB (20/27), and none showed any evidence of an association with a pain phenotype. This likely reflects weaker evidence of association for SFN NaV1.7 mutations, with no family pedigree indicating heritability and no statistically significant association with painful neuropathy. Accordingly, it is probable that some Na_V_1.7 mutations have been mislabelled as pathogenic and that causal mutations in other genes have likely been missed. In two previous erythromelalgia cohort studies, only 15% (2/7) and 28% (7/48) of patients had a rare Na_V_1.7 mutation, and all of the mutations observed within the cohorts had been previously reported in other erythromelalgia studies suggesting that we are approaching saturation in the discovery of new causal mutations.^18,60^ Given these findings, it is increasingly likely that many cases of erythromelalgia are not due to gain-of-function Na_V_1.7 mutations. While some mutation carriers may possess protective variants influencing the pain phenotype, this alone cannot explain the lack of an increased risk of chronic pain in carriers of penetrative mutations that should render sensory neurons hyperexcitable.^75,76^ Of the two reported modifier mutations of the erythromelalgia phenotype, *KCNQ2* T730A (gnomAD v4.1.0 MAF<0.00001) and *KCNQ3* D755N (gnomAD v4.1.0 MAF=0.0005), the former is not present in UKB, whereas the latter has 527 carriers. Based on prevalence alone, *KCNQ3* D755N cannot account for the lack of penetrance in the many carriers of pathogenic VGSC mutation present in UKB. Furthermore, being a *KCNQ3* D755N carrier does not reduce the risk of chronic pain compared with the rest of the UKB cohort (Fisher’s exact test, OR = 1.05 [0.88–1.25], *P* = 0.57).^75,76^ Marked intra- and inter-family variability in phenotypic expression of NaV1.7 gain-of-function mutations has been documented; however, this does not preclude detection of increased pain risk at the population level.^59^

Our findings suggest many VGSC mutations are not pain-pathogenic in humans, and gain-of-function may be limited to *in vitro* models. This could be due to a relative overexpression of the transfected VGSCs, the absence of normal regulatory interacting proteins, abnormal distribution of proteins within cell lines and alterations to cellular phenotype from the culture conditions or differentiation protocol. The typical discovery sequence for a potentially pathogenic VGSC mutation from clinical phenotyping in rare pain patients usually precedes electrophysiological studies which are then conducted in the expectation that there is likely to be a gain-of-function in some aspect of channel biophysics. We note that only one electrophysiological study from those reviewed presented negative findings from functional testing of potential erythromelalgia mutations suggesting the presence of positive publication bias.^77^ These electrophysiological findings are seldom modelled within a nociceptor to determine whether altered conductance can account for increased excitability or spontaneous activity. Furthermore, these variants are rarely placed back into the *in vivo* context. Where that has been done, for example mouse or zebrafish models of the Na_V_1.7^I228M^ mutation, they do not recapitulate the erythromelalgia phenotype.^63,78,79^ Perhaps these mutations do not cause the same gain-of-function *in vivo* that they do *in vitro*, or maybe they do cause a degree of hyperexcitability of DRG neurons, but this is insufficient to cause pain. The field will need to be more stringent in its appraisal of *in vitro* electrophysiological findings which have hitherto been regarded as the gold-standard evidence for identifying the gain-of-function in pathogenic VGSC mutations.

The VGSC mutations that were either not observed in UKB at all or were excluded from analysis because they had <50 carriers, are more likely to be pathogenic due to their rarity. Of the 58 pathogenic Na_V_1.7 mutations reported in the literature, 39 were so rare that they were not observed in UKB, nor can they be found in the gnomAD database (v4.1.0). There was a substantial difference in the average CADD, AlphaMissense and EVE pathogenicity scores of VGSC mutations observed versus absent from UKB (only EVE does not use MAF as either a model feature (CADD) or for fine-tuning [AlphaMissense]).^43,80^ Another method to triangulate pathogenicity predictions for a specific amino acid change is through comparative functional analysis of paralogues in other human VGSCs.^4^ None of the putative pathogenic Na_V_ mutations in UKB had any corroborating electrophysiological evidence, whereas almost one-third of the mutations absent from UKB had electrophysiologically-confirmed paralogues. This was also mirrored in the counts of paralogues with inherited disease associations (such as epilepsy, long QT syndrome and myotonia) with only 36% (in UKB) versus over 90% (absent from UKB). This can also be seen in the distribution of the rarest mutations within the VGSC protein structure which are concentrated in the most conserved and functionally important domains such as the S4 voltage sensor, the fast inactivation gate in D3–D4 linker and in the pore domains S5 and S6 unlike that seen in the commoner mutations.

Little research has been undertaken in heterozygous loss-of-function VGSC mutation carriers. We found this to have no impact on any pain phenotype we assessed. One proposed explanation for this is that heterozygous (theoretically 50%) loss-of-function of Na_V_1.7, Na_V_1.8 or Na_V_1.9 does not reduce protein expression enough to lead to altered neuronal excitability. This aligns with functional evidence that suggests around an 80% reduction in conductance would be required to provide analgesia.^81^ While no phenotypic associations were observed for any of the heterozygous predicted loss-of-function carriers, we observed two participants with a homozygous loss-of-function in Na_V_1.9. The mutations were W1005* and G168Sfs*11, which both result in a premature stop codon, the latter 11 amino acids after a frameshift. To our knowledge, these are the first reports of humans with a full Na_V_1.9 loss-of-function. They both reported having chronic pain at recruitment.

One potential limitation of the use of UKB for this study is that it is a volunteer population cohort that recruited ∼6% of the 9 million approached and this led to a known bias towards healthier, more affluent and white population limiting its representativeness.^82^ Importantly in this context, the age at recruitment was 37–73, whereas erythromelalgia typically first presents in childhood and PEPD in infancy. This could have reduced the number of patients with inherited pain conditions as they would be less likely to enrol, which one would expect to reduce the prevalence of causal VGSC mutations. The comparison to MAFs from gnomAD v4.1.0 non-UKB is therefore pertinent; however, the MAFs for these VGSC variants are similar. This recruitment bias cannot explain why many putative pathogenic VGSC mutations are relatively common in UKB, nor their lack of effect on pain phenotypes. While some of the SFN variants are considered risk factors rather than causal for disease nonetheless the age range of the UKB cohort should have been sufficient for any delayed phenotype to manifest as a pain condition.^83^ While our analysis would detect both increases or decreases in the risk of each phenotype, the complex relationship between channel hyperexcitability and pain is difficult to disentangle. For example, a paradoxical loss-of-function can be observed due to a large gain-of-function in channel excitability.^84,85^

There is a prior report of an association of a ‘Neanderthal’ Na_V_1.7 haplotype with pain.^57^ Inheritance of three mutations (M932L, V991L and D1908G) in tight linkage disequilibrium was associated with an increased prevalence of a composite pain phenotype in UKB. This haplotype was excluded from our study of single gain-of-function mutations, but we replicated this analysis using our pipeline and find that there are 2243 carriers in the UKB 500k WES dataset. With our Na_V_1.7 control population, which excludes carriers of other putative gain-of-function mutations, we observed an OR of 1.11 [1.02–1.21] for chronic pain—lower than the previous estimate of 1.16 based on a smaller population of genotyped individuals (1327).^57^ This association does not survive correction for multiple comparisons (*P* = 0.12), nor are any of the other outcomes significant, suggesting that it is not strongly associated with a population-level chronic pain phenotype in the full UKB dataset (Supplementary Fig. 1, Supplementary Table 10).

From a clinical diagnostic perspective, the Association for Clinical Genomic Science recommends using family history, primary literature, gnomAD MAF and public databases, such as Ensembl and ClinVar, to determine pathogenicity.^86^ Hence familial disease segregation of a rare variant that causes a missense at an amino acid residue with strong evolutionary conservation alone is insufficient to confirm pathogenicity. While *in vitro* functional evidence of gain-of-function improves the rationale for attributing pathogenicity to a mutation; our observations highlight a discordance between *in vitro* functional evidence and the lack of an increased prevalence of chronic pain observed in carriers of these mutations in UKB.

Nearly all previous Na_V_1.7–1.9 mutations have been identified by targeted sequencing of the respective genes in patients with a chronic pain condition. By identifying carriers of these previously reported pathogenic mutations, we have been able to flip the question typically asked in case studies to see if these mutations are associated with pain. In contrast to the existing literature, almost half of these putatively pathogenic mutations show no association and do not confer even a modest pain predisposition. As a consequence, other potential causal genetic variants and modifier genes have likely been missed. The pain genetics field would likely benefit from pooling data from large consortia of clinicians, geneticists and electrophysiologists to identify which of the VGSC mutations are truly causal for pain (similar to that for cystic fibrosis https://cftr2.org). Banked patient DNA could be exome- or genome-sequenced to reassess causal links. Besides forming a useful resource for the patient, clinical and pain research community, it could also provide a point of access for investigators to recruit channelopathy patients for clinical trials—this is currently quite challenging due to their rarity and has likely contributed to the small number of therapeutic clinical trials for this disease group.

## Supplementary Material

fcag374_Supplementary_Data

## Data Availability

The outputs of the analysis are provided in the paper and supplementary materials. Researchers can access the data from UKB by applying at https://www.ukbiobank.ac.uk/enable-your-research/apply-for-access. The annotated code to process the genomics and phenotype data on the UKB RAP and perform the full analysis in R, including generation of all visualizations presented in this manuscript, is available at https://github.com/graemenewton/Newton-2026-NaV1.x-Pain-UK-Biobank.
