# Supplementary material for: NaV1.7, 1.8 and 1.9 mutations linked to painful channelopathies have no pain phenotype in UK Biobank
Source: Brain Commun. 2026 Oct 7;8(5):fcag374. doi: 10.1093/braincomms/fcag374 (PMC13684400; doi:10.1093/braincomms/fcag374)
Supplement: Supplementary file 1 [file fcag374_supplementary_data.zip › Supplementary_material.docx]

Na_V_1.7, 1.8 and 1.9 mutations linked to painful channelopathies have no pain phenotype in UK Biobank

Graeme W.T. Newton^1,2^, Corin Charman^1,3^, Aidan P. Nickerson^1,3^, Keith G. Phillips^1,4^, Achim Kless^3^, James P. Dunham^1^ and Anthony E. Pickering^1^

# Supplementary Material

**Supplementary Figure 1** The Na_V_1.7 Neanderthal haplotype is not associated with increased risk of chronic pain, neuropathic pain, or analgesic or anti-neuropathic medication prescriptions. Odds ratios for each phenotype are presented with their 95% confidence intervals. TCA = Tricyclic Antidepressant.

**Supplementary Table 1** Data fields used to generate the chronic pain phenotype group. ORA = Original Recruitment Assessment.

**Supplementary Table 2** Data fields used to generate the neuropathic pain (DN4) phenotype group. EoPQ = Experience of Pain Questionnaire.

**Supplementary Table 3** Analgesic prescription phenotype groups and the regular expression patterns used to extract prescription records for each class of drug.

**Supplementary Table 4** List of multi-carriers excluded from the main analysis for each gene model.

**Supplementary Table 5** – Logistic regression model summaries for the effects of mutations on the risk of chronic pain, neuropathic pain, gabapentinoid, Na_V_ blockers, dual mechanism opioids, strong opioids, and tricyclic antidepressants.

**Supplementary Table 6** List of Na_V_1.7, Na_V_1.8, and Na_V_1.9 gain-of-function channelopathy mutations, their disease association, and associations of their paralogue mutations. Data on paralogue electrophysiology and disease associations is also included for triangulation (<https://scn-viewer.broadinstitute.org/>).^1^ Variant ID presented in vcf file format: *“chromosome: position: reference allele: alternative allele”.* Ephys GoF = Electrophysiology Gain-of-Function. EM = Erythromelalgia, PEPD = Paroxysmal Extreme Pain Disorder, FEPS = Familial Episodic Pain Syndrome, SFN = Small Fibre Neuropathy, EP = Epilepsy.

**Supplementary Table 7** List of all predicted loss-of-function VGSC mutations that were grouped on the gene-level.

**Supplementary Table 8** Number of carriers, minor allele frequency (MAF), electrophysiology gain-of-function evidence status, and disease association from case report. This table includes UniProt disease associations, machine learning pathogenicity prediction scores, and intra- and inter-species conservation for the putative pathogenic Na_V_1.7, Na_V_1.8, and Na_V_1.9 channelopathy mutations observed in UKB. * = originally proposed as an EM mutation but now considered to be SFN. † = mutation caused by 2 unique genomic variants. ‡ = mutation not considered as pathogenic for this analysis due to commonality. Het = number of heterozygous carriers, Hom = number of homozygous carriers, UKB MAF = UK Biobank MAF, 1000G = 1000 Genomes Project MAF, gnomAD = gnomAD 4.1 non-UKB MAF, Ephys GoF = electrophysiological gain-of-function status, AM = AlphaMissense score, EVE = EVE score, CADD = CADD score, Inter = interspecies Na_V_1.7 sequence alignment score, Intra = intraspecies human Na_V_ family protein alignment. EM = erythromelalgia, PEPD = Paroxysmal Extreme Pain Disorder, EP = Epilepsy, SFN = Small Fibre Neuropathy.

**Supplementary Table 9** – Logistic regression model summaries for the combined rare gain-of-function model (carriers of any mutation with <50 carriers vs everyone else) and all gain-of-function (any carrier of any putative pathogenic mutation, including multi-carriers, vs everyone else) against the chronic pain phenotype. *P* values presented in this table are unadjusted for multiple comparisons.

**Supplementary Table 10** – Logistic regression model summaries for the Na_V_1.7 Neanderthal haplotype analysis (M932L, V991L, D1908G) on the risk of chronic pain, neuropathic pain, gabapentinoid, Na_V_ blockers, dual mechanism opioids, strong opioids, and tricyclic antidepressants.

# Supplementary References

1.	Brunklaus A, Feng T, Brünger T*, et al*. Gene variant effects across sodium channelopathies predict function and guide precision therapy. *Brain*. 2022;145(12):4275-4286. doi:10.1093/brain/awac006
